# Challenges and opportunities in the network medicine of complex diseases

**DOI:** 10.1016/j.medj.2025.100920

**Published:** 2025-11-20

**Authors:** Valeria d’Andrea, Joseph Loscalzo, Manlio De Domenico

**Affiliations:** 1 Department of Physics and Astronomy “Galileo Galilei,” University of Padova, Padova, Italy; 2 Department of Medicine, Harvard Medical School, Boston, MA, USA; 3 Channing Division of Network Medicine, Brigham and Women’s Hospital, Boston, MA, USA; 4 Padua Center for Network Medicine, University of Padova, Padova, Italy; 5 Padua Neuroscience Center, University of Padova, Padova, Italy

## Abstract

Network medicine applies fundamental principles of complexity science and systems medicine to integrate and analyze complex structured data, including genomics, transcriptomics, proteomics, and metabolomics, to characterize the dynamical states of health and disease within biological networks. In this perspective, we discuss the major successes of the field and how incorporating techniques based on statistical physics and machine learning in network medicine has significantly refined our understanding of disease networks. Despite these achievements, the maturation of network medicine presents challenges that must be addressed. Limitations in defining biological units and interactions, interpreting network models, and accounting for experimental uncertainties hinder the field’s progress. The next phase of network medicine must expand the current framework by incorporating more realistic assumptions about biological units and their interactions across multiple relevant scales. This expansion is crucial for advancing our understanding of complex diseases and improving strategies for their diagnosis, treatment, and prevention.

## INTRODUCTION

Network biology is a discipline that utilizes network-based approaches to study complex biological systems. This relatively new field involves the use of mathematical and computational techniques to model and analyze complex multidimensional biological entities, such as (systems of) genes, proteins, or metabolites, both statically and dynamically. By representing those biological molecules as nodes and their interactions as edges, network biology provides a powerful framework for understanding the structure, function, and dynamics of biological systems (see Box 1 for an introduction to basic concepts in network science, with a focus on systems medicine and biology). The principal aims of network biology include the integration and classification of the intricate networks that govern cellular processes, signaling pathways, and molecular interactions and the exploration of how interactions between individual components give rise to emergent properties at the systems level, uncovering nodes and edges within networks that play pivotal roles in biological functions.^1,2^ Network medicine derives from network biology and leverages the complex interactions between genes, proteins, and other molecular components within biological systems to gain insights into disease biology. An illustrative application of this is the focus on genetic variants and mutants that alter the abundance or function of their protein products to perturb the structure or dynamic behavior of the subnetwork (i.e., specific disease module) within which the gene product operates. This conceptual framework underlies several studies reviewed in this paper, which illustrate how disease-associated genetic variants can perturb protein-protein interaction (PPI) networks, disrupt disease modules, and ultimately contribute to complex pathophysiological mechanisms (see clinical applications of network medicine). The aims of network medicine encompass uncovering unknown relationships that elucidate disease mechanisms and identifying biomarkers that can be used to diagnose diseases, monitor disease progression, and predict treatment response. The purposes of network medicine also include the design of targeted therapies through the identification of molecules and interactions that may be pivotal in the disease network, and advancing precision medicine by taking into account individual specificity in genes and the environment to optimize therapeutic outcomes.^1,3–5^

## INSIGHTS FROM NETWORK MEDICINE

In recent years, multiple successes have been achieved in both the fields of network biology and network medicine, providing insights into the complex interactions and relationships between molecular components within biological systems,^13,27,28^ identifying disease modules and pathways among distinct diseases,^3,29^ predicting drug-disease interactions,^30^ and defining the phenotypic effects of genetic variations and mutations.^31^ Those successful applications have been made possible by overcoming the approach adopted in traditional biomedical research that embraces a reductionist stance, concentrating on pinpointing a handful of altered components—genes, proteins, or metabolites—that directly contribute to a particular disease. Nonetheless, comprehending the function of any one element in biological systems often requires examining its interactions within the larger system. This integrative, holistic approach is where network medicine emerges, drawing upon network theory and biomedical data.

### Clinical applications of network medicine

#### Understanding disease mechanisms

There are many examples of the success of network medicine as an approach to understanding disease complexity. A central premise of network medicine is that genetic mutations can perturb the structure and function of molecular interaction networks, thereby driving disease mechanisms. In this context, network-based representations shift the focus from individual genes to their roles within dynamic, interconnected systems. Network medicine informs genetics, as demonstrated by the significantly increased frequency of disease-causing germline and somatic mutations occurring at protein-protein binding interface sites compared with non-interface sites.^32,33^

This observation reflects a broader insight of network medicine, i.e., that the functional impact of mutations is often determined not only by the affected gene itself but also by its topological context, such as its connectivity and network neighborhood. A variety of node-level metrics, such as degree and betweenness centrality, can be computed and provide key micro-scale analytical insights. These approaches, further elaborated in the following sections, highlight the role of highly connected or central nodes whose disruption is more likely to result in disease phenotypes due to the propagation of their effects across the system.^21,34^

Modular analysis is also crucial. Many biological networks display a community or modular structure, where groups of genes or proteins are more densely interconnected with each other than with the rest of the network. These modules are not isolated from each other: they interact and frequently overlap.

Uncovering disease modules has relied on various computational approaches, from mesoscale analyses that identify community structures to macro-scale methods that trace system-wide information flow. Together, these complementary strategies reflect the multilevel complexity of disease networks (see integrative multi-scale frameworks in network medicine).

In the context of disease, identifying such modules has been instrumental in revealing shared biological processes across distinct conditions. Network medicine approaches have also been used to identify endophenotype modules, which comprise pathways that govern basic (generic) disease pathogenesis, including inflammation, thrombosis, fibrosis, and autoimmunity,^35,36^ and molecular pathways shared across apparently unrelated diseases.^37^

Module identification helps address clinical challenges, such as the heterogeneity of disease presentation and the lack of reliable biomarkers. In complex diseases such as cardiovascular or neurodegenerative disorders, identifying shared or distinct molecular modules aids in both patient stratification and personalized therapeutic strategies.^2,3^ Thus, these approaches not only deepen our understanding of disease mechanisms but also translate into tangible clinical benefits by guiding diagnostics and interventions.

#### Biomarker discovery and patient stratification

Network medicine approaches have been instrumental in discovering potential biomarkers for a wide range of complex conditions, including autoimmune diseases, cancer, and infectious diseases.^3^ By modeling diseases as interconnected networks of genes, proteins, and other molecular entities, network medicine enables researchers to identify key nodes and pathways that drive disease mechanisms. For example, tools that leverage PPI networks have been recently developed to prioritize biomarkers and predict patient responses to treatments for rheumatoid arthritis and ulcerative colitis, significantly enhancing the precision of therapeutic interventions.^38^

Building on these advances, network-based frameworks have also been used by clinicians to better predict disease risk and tailor treatments to individual patients, solidifying their role as a cornerstone of precision medicine. These approaches represent a transformative advance, enabling the integration of diverse datasets, including genomic, transcriptomic, and proteomic profiles, to build predictive models that are highly adaptable to individual variability.

For example, the development of individualized PPI networks has provided unique insights into patient-specific pathobiology, such as in hypertrophic cardiomyopathy (HCM), where tailored interactomes have been used to identify critical and distinctive pathways influencing disease expression and outcome.^39^ Similarly, in the context of cancer, shared molecular signatures derived from multi-cancer interaction networks have demonstrated strong associations with patient mortality, enabling better stratification of risk and targeted therapeutic strategies across diverse cancer types.^40^

Moreover, network medicine approaches have shown promise in complex diseases beyond cancer. For example, cardiovascular diseases and pulmonary arterial hypertension have benefited from network analysis by identifying critical molecular nodes, such as fibrosis-specific subnetworks, that drive disease progression and serve as therapeutic targets.^41,42^ Additionally, network medicine has proven effective in dissecting the molecular underpinnings of diseases to redefine them based on endotypes—distinct molecular mechanisms rather than traditional phenotype-based classifications. This shift allows for identifying biomarkers more closely tied to disease mechanisms, enabling personalized treatments. For example, analyses using disease-disease networks and multi-omics datasets have successfully uncovered molecular signatures that distinguish subtypes of conditions such as type 2 diabetes and neurodegenerative disorders.^43,44^

#### Drug discovery and repurposing

Network medicine approaches have also shown great potential in advancing drug discovery and repurposing by leveraging molecular interaction networks to identify novel therapeutic targets and repurpose existing drugs efficiently. Traditional drug discovery often relies on the “one-disease, one-target” paradigm, which fails to address the complex, interconnected nature of biological systems. Network-based methodologies offer a transformative alternative by focusing on disease modules, i.e., subnetworks of interconnected genes or proteins implicated in specific pathological states. These approaches allow the identification of potential drug targets and therapeutic interventions at a systems level, fostering innovation in treatment strategies and meeting the challenges posed by multifactorial diseases.^5,45,46^ For example, network medicine has been successfully applied to repurposing drugs for complex diseases such as Alzheimer disease, where single-cell network biology integrates transcriptomic and chromatin interaction data to identify regulatory networks and prioritize candidate drugs. Such frameworks can predict drug-disease relationships with high accuracy and facilitate validation of these predictions experimentally.^47^ Similarly, during the COVID-19 pandemic, network diffusion and proximity algorithms, as well as network-based AI methods, were utilized to map SARS-CoV-2-host interactions, leading to the potential for repurposing several clinically approved drugs with substantial efficacy against the virus. This methodology significantly outperformed traditional screening approaches in both speed and success rate.^5,48^ Moreover, network pharmacology has demonstrated its utility in repurposing drugs for coronary artery disease^32^ and vascular calcification,^49^ as well as in identifying synergistic drug combinations. For example, in ischemic stroke, protein-metabolite interaction networks were used to identify NADPH oxidase and nitric oxide synthase as synergistic targets. Experimental validation confirmed the enhanced efficacy of targeting these nodes together, offering a powerful example of how network medicine can guide combination therapies to address unmet medical needs.^50^ These developments closely align with the field of network pharmacology, which further extends the principles of network medicine by explicitly modeling the complex, multi-target nature of drug-disease interactions. Network pharmacology employs integrative computational workflows to explore the pharmacological landscape systematically.^51,52^ These approaches have been successfully applied to the identification of synergistic drug pairs and to the repurposing of existing compounds for diseases such as cancer, diabetes, and inflammatory disorders. By accounting for the systems-level behavior of drug actions, network pharmacology helps address key clinical limitations of traditional drug discovery, including off-target toxicity and therapeutic resistance, ultimately contributing to more precise and effective interventions.^53^

### Integrative multi-scale frameworks in network medicine

Multiple approaches formally developed by network theory have enabled the achievement of these results. A first logical division of such methods can be made by considering the different scales used to characterize the phenomenon under study (see Figure 1). Micro-scale approaches account for features at the level of individual network nodes. Centrality measures, for example, have been utilized to identify which units hold greater importance in the functioning of the network.^21,54^ At a larger scale, mesoscopic descriptors identify the presence of functional modules or communities among the network units, which are fundamental for describing its segregated-while-integrated functioning. Common approaches include community detection algorithms such as Louvain and MCL (Markov clustering algorithm) for non-overlapping modules and k-clique percolation for overlapping structures that reflect protein multifunctionality.^14,34,55–57^ Measures that quantify the flow of information within the network characterize the system on a global (or macroscopic) scale. These quantities have significance in terms of generalized thermodynamics^58^ and are used to understand and amplify the impact of perturbations (such as gene mutations or interactions with viral components) on the system.^59–61^ From a computational perspective, methods for identifying drug targets that utilize network structure coupled with machine learning,^62^ AI,^63^ and near-exascale computing of drug-target interactions in association with physicochemical features of the drug compounds^64^ have each met with success and continue to improve upon the predictive accuracy of prior methods that have not incorporated network topologies. Lastly, the nascent growth of quantum computing offers the promise of advancing network medicine further with greatly accelerated computational speed, quantum-inspired computational algorithms (e.g., disease module identification^65^), and improved performance.

To capture the complexity of interacting systems within a unified framework, it is, indeed, necessary to describe them as networks of networks, or multilayer networks. This approach, capable of capturing interactions among different systems, has yielded numerous successful applications in the modeling and analysis of empirical biological systems, particularly in the field of network medicine. For example, various interdependent systems, such as the genome, proteome, and metabolome, can be represented as different layers, which take into account the relationships among the constituent units of such systems. This integrated approach, known as multi-omics, has proven to be valuable in a broad array of applications, including, but not limited to,^66–68^ the classification of complex diseases,^54^ the identification of disease-relevant modules,^57^ and the description of the molecular mechanisms responsible for the pathogenesis of (non-)Mendelian diseases.^69,70^ More generally, networks composed of more than one type of node and enabling the integration of heterogeneous data are referred to as multipartite networks. Typical examples in systems biology include gene regulatory networks, where nodes represent regulators and target genes, or networks formed by PPIs, disease genes, and drug targets (see Lee and Loscalzo^67^ and Jafari et al.^71^). This type of modeling has facilitated a range of successful applications in the field of network medicine, such as the development of network-targeted therapies^72^ or the identification of new patterns linking viral infections to diseases.^73^

Lastly, the ability to resolve biological processes at specific spatial and temporal scales offers new opportunities to deepen our understanding of disease mechanisms. Spatiotemporal omics provides a comprehensive framework for understanding biological systems by integrating molecular measurements with spatial localization and dynamic temporal changes. This approach enables the mapping of cellular composition, gene expression, and cell-cell interactions across tissues and developmental time, offering critical insights into processes such as morphogenesis, immune organization, and disease evolution.^74,75^ In contrast, single-cell omics focuses on profiling molecular features, such as transcriptomes, epigenomes, or proteomes, at the resolution of individual cells, independent of their spatial or temporal context. This framework has been widely applied to uncover cellular heterogeneity, lineage trajectories, and cell states in complex systems.^76^ For instance, single-cell transcriptomics has provided markers of early malignant transformation in gastric cancer,^77^ while integrated single-cell and spatial omics have revealed the structure and development of human thymocytes.^78^ To fully exploit these frameworks, advanced computational methods are essential. Statistical models and machine learning techniques can be critically important for capturing spatial and temporal dependencies, reconstructing cell states, and inferring regulatory networks from high-dimensional, multi-omic data at single-cell or spatial resolution.^74,76^

## CURRENT CHALLENGES FOR NETWORK MEDICINE

Despite the aforementioned successes, the development of network medicine as a branch of network science carries some caveats and challenges that we must be aware of and that should be overcome for the full development of the field. Network science is, indeed, a relatively new discipline, with a strong mathematical and physical theoretical foundation (see Box 2 for a list of key network definitions relevant to network medicine). It is important to be aware of the assumptions used when collecting experimental data, in their subsequent representation, and when choosing appropriate methods for their analysis in order to guarantee more successful applications and reproducible results. The simplest definition of a network is that of a system constituted by fundamental units, known as nodes, and their interactions, referred to as links. However, establishing the connection between this abstract definition and the real system under study is not trivial.

### Limitations in the definition of biological units

Let us consider the definition of nodes within a biological system of interacting proteins. It is, of course, always imperative to bear in mind the incompleteness and uncertainty of our measurements. When studying an interactome, for example, the percentage of known proteins is highly variable and heavily dependent on both the organism under study and the experimental methods employed to identify and characterize proteins. It has been estimated that, despite significant advances in mapping the interactome through high-throughput methodologies, such as yeast two-hybrid screening and various literature-curated data repositories, the current state of interactome mapping remains largely incomplete^4,37^ (see Figures 2A and 2B). Even when focusing on the known nodes and despite considering the groundbreaking successes of AlphaFold, the internal structure (e.g., protein tertiary structure) of the nodes themselves remains a feature for which we can only express a level of confidence.^79^

Furthermore, the same attributes or metadata associated with individual nodes may be subject to error. The impact of this type of uncertainty is particularly significant when matching data from various data sources, i.e., when integrating two or more datasets collected independently with different modalities. This challenge has been exemplified in recent years in the development of effective multi-omics approaches.^68,80–84^

The selection of variables to be described as nodes in the network is of crucial importance. In the standard approach for PPI networks, for example, proteins are typically modeled as geometric points,^16,85^ and no explicit assumptions about their internal structure are included in the model. Nevertheless, a more accurate model could entail a physicochemically enriched network framework, in which nodes represent physical proteins, that explicitly takes into account relevant protein features (see Figure 2C). To provide some examples, these features might include domains in multi-domain proteins, regarded as autonomous folding units and functional/evolutionary units of the protein.^86,87^ Alternatively, a relevant subset of residues, such as the protein’s (enzyme’s) active site and/or PPI interface, could be considered. In this framework, a network should encompass not only proteins but also interacting ligands or drugs and their physicochemical features^64^ or, more broadly, any substrate capable of interacting with them and inducing structural and functional changes to the network through pairwise interactions, such as allosteric mechanisms. In addition and importantly, the PPI as currently conceived fails to take into account two additional limitations of note. First, each PPI is thermodynamically governed by a binding constant reflecting the free energy of association. The fraction of protein molecules bound in the pairwise complex then depends not only on this binding energy but also on the intracellular concentrations of each protein (see Figure 2E). Second, the binding of one protein to another is generally defined by crystal structure analysis of the protein-protein complex, which reflects a single instantiation of the proteins’ tertiary structures (i.e., those that are co-crystallizable). As cryo-electron microscopy studies show, however, there are often many different conformations available to a protein (in solution). Taken together, these limitations imply that the true PPI is an ensemble of states, the relevant dynamic behavior of which depends on the timescale under consideration and other environmental and epigenetic perturbations (e.g., temperature, pH, and post-translational modifications). Such a physicochemically enriched network offers the advantage of describing, within a unified framework, interdependencies across different spatial scales that range from those of individual proteins to interactions between them and is more realistically suitable for capturing the effects of perturbations propagating across multiple scales.

### Limitations in the definition of interactions

Not only nodes but also links can be prone to errors and uncertainties owing to the reconstruction of interactions based on correlation, thresholding, or spatial proximity methods. A typical example can be observed in gene co-expression networks (GCNs), where pairs of nodes, each representing a single gene, are linked if they exhibit a similar expression pattern in response to an external stimulus or condition.^88,89^ Various methods have been developed for GCN reconstruction, but the general approach typically involves two steps: a co-expression measure, usually a correlation measure such as Pearson or Spearman rank correlation, as well as mutual information, followed by a thresholding procedure so that gene pairs with a co-expression measure higher than the selected threshold are connected by an edge in the network. A similar approach is used to describe functional connectivity measures among brain regions, which are evaluated with correlations between changes in, e.g., the blood-oxygen-level-dependent (BOLD) signal due to changes in neural activity over time.^90^ Other examples include residue interaction networks (RINs), where nodes representing residues are connected if they are in close proximity in the protein tertiary structure,^91,92^ and interacting protein networks colocalized to the same cellular compartment. In the aforementioned examples, the reconstruction of network links is susceptible to errors due to both a lack of robust choice of statistical similarity descriptors and an absence of objective criteria to threshold the resulting similarity matrices for filtering the observed correlations.^93^

The issue of uncertainty in network reconstruction has been addressed by some studies in the biological sciences, such as those focusing on gene regulatory networks^94^ or PPI networks.^95,96^ However, much remains to be done.

### Limitations in interpretability

Another aspect related to network descriptor estimates that requires careful consideration is the oversimplification of the null models used to test hypotheses. For example, there is the assumption that disease-associated proteins interact more frequently with each other compared to randomly selected proteins and aggregate in specific regions of the interactome, suggesting the existence of specific disease modules for each disease.^97^ Community detection algorithms used to identify these modules often exhibit deviations from random null models, but in the case of multidimensional systems such as interactomes, simple random null models are not biologically plausible.

Similar considerations to those required for the analysis of structural complexity must also be taken into account for the assessment of the dynamical complexity of many biological processes. Processes such as gene regulation, transcription, translation, and reaction kinetics in metabolic networks must be described using time-series measurements that occur superordinate to the static structure of (a subset of) the nodes of the reconstructed network, with a dynamics that is highly nonlinear and non-uniform across the nodes due to heterogeneity in the initial conditions and of the underlying networks and subnetworks. In this context, it is crucial to consider dynamics that are biologically plausible and that have been found to describe many biological contexts in the time domain. An example is provided by the Michaelis-Menten law, which describes enzyme behavior, in general, and whose ubiquity has been shown to derive from the fulfillment of simple, appropriate structural network conditions.^17,98,99^

## CONCLUSION

Network biology and systems medicine have succeeded in demonstrating how the network paradigm can help to better understand the complex processes that characterize complex biological systems. Despite the fact that the field is still in its infancy, its potential for becoming the reference framework for precision medicine is increasingly acknowledged. We identify the following fundamental steps needed to catalyze a second network medicine revolution.

### Move beyond isolated components or sub-systems toward interdependent and multilayer models

Comprehending the function of any one element in biological systems often requires examining its interactions within the larger system. While network medicine is inherently non-reductionist,^100^ its current framework is still often based on modeling and analyzing a sub-system (e.g., a cell or a circuit) separate from other sub-systems and the organism as a whole. To some extent, even a more extended framework that models the interdependency between dynamics at the level of populations of organisms and the systems biology at the individual level is desirable and aligned with One Health and Global Health goals.^101^ A more comprehensive perspective, integrating different sub-systems, is needed to enhance our understanding of a complex biological system. This is an ambitious goal and requires the design of novel experimental setups and data gathering to capture not only the structure and dynamics within each sub-system but also the structure and dynamics across distinct sub-systems (see Figure 2F). The mathematical framework needed to achieve this goal is sufficiently mature^26^ and beginning to show its descriptive and predictive power.^25,102–104^ The same framework has been used to better characterize the robustness and the resilience of complex physical systems to perturbations.^105^ It is also important to point out the typical aspects of interactions among biological systems that are not fully captured by a multilayer approach. Among these, it is pertinent to mention the integration of dynamics across different scales, aimed at understanding how processes occurring at a specific level, for example, metabolic processes, can influence dynamics at a higher level, such as functional cellular dynamics. Integrating processes across different scales is challenging both because they are often described by laws of different natures and because biological systems are usually characterized by emergent phenomena, which cannot be deduced from a description, albeit comprehensive, of phenomena occurring at a lower scale.^26^

### Embrace experimental and statistical uncertainty in network reconstruction and analysis

Uncertainty must be embraced, and methods that explicitly address inherent experimental error measurements and the existence of false positives/negatives in the interaction among the biological units of interest must be correctly taken into account. This ambitious goal can be achieved by designing novel experimental setups able to capture more complex information about those units and their interactions and by moving beyond the correlation/similarity paradigm to include, from the outset, generative models of plausible physicochemical and biological origins for both the structure and the dynamics of the intervening determinants. In recent years, a promising approach in real-world applications of network science has emerged that involves recognizing the inherent level of error in network reconstruction.^106^ This recognition can be accomplished through statistical inference methods, by the estimation of the existence of each edge via repeated measurements,^107^ and by considering sets of generative models that could potentially give rise to the observed network^108,109^ (see Figure 2D). In addition, a Bayesian approach can be adopted, resulting in an ensemble of possible networks that incorporates the uncertainty arising from the measurements and can also be applied when the network data have been measured only once.^110^ It is of paramount importance to recognize and remark that uncertainties also extend to and are reflected in the estimation of network descriptors, which, in such approaches, are not represented by a single estimate but rather by a stochastic variable requiring a probability distribution for description.^106,111^

### Enhance network maps with generative models and more biologically relevant assumptions

The conceptualization of a healthy living organism as a steady dynamical state of a complex system, encompassing physical, chemical, and biological processes out of equilibrium, has provided a novel framework for understanding disease. Within this paradigm, diseases are seen as perturbations of the network’s non-equilibrium steady state, leveraging the principles of dynamical systems to offer insights into the stability and resilience of biological networks. This perspective enhances our understanding of the onset, progression, and potential reversal of pathological states. A better mapping of the physical, chemical, and biological processes that can be used for the control^112^ of empirical biological systems is needed in order to bring them to a desired *healthy state* by targeted external interventions and controlled perturbation.^113^ The current development of a framework for allowing this controlled behavior is established: from the early detection of warning signals of network dysfunction^114^ to strategies for limiting the spread of functional failures^115^ and enhancing network recovery.^18^ Achieving this goal optimally will require precise determinations of intracellular concentrations of proteins, metabolites, enzymatic rate constants, and protein-protein association constants, the latter two being vastly different in the protein-rich intracellular (and intracompartmental) milieu than in dilute solutions of purified proteins, in which these measurements are typically made (cf. Elgart et al.^116^ and Zotter et al.^117^).

As network medicine continues to evolve, it stands at the frontier of a major paradigm shift in how we understand and treat complex diseases. This discipline, grounded in the principles of network science, offers a comprehensive framework for integrating diverse biological data and computational models, thereby enhancing our understanding of the multifaceted nature of disease mechanisms. The promise of network medicine and its desirable future developments lies in its potential to transcend conventional analytical approaches by explicitly taking into account interconnections and interdependencies that characterize biological systems. The path forward involves not only refining these models to handle the complexity inherent in biological data but also ensuring that such models can be effectively translated into clinical settings. This approach is poised to revolutionize precision medicine by facilitating the development of treatments that are finely tuned to the individual characteristics of each patient’s disease process. As such, network medicine could become indispensable in clinical practice, driving innovations that extend from diagnostic processes to therapeutic interventions and preventive measures. Embracing this approach will likely yield significant advances in our capacity to manage and cure diseases, highlighting the transformative impact of integrating network science into healthcare. This strategic integration promises a future where medicine is not only personalized but also more predictive, preventive, and precise.

## Figures and Tables

**Figure 1.**
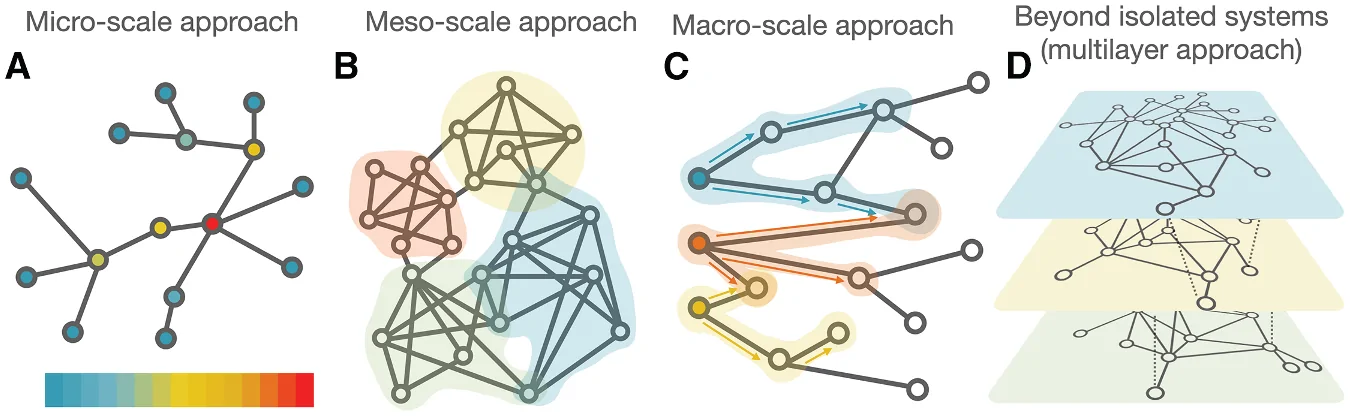
Main analytical approaches in network medicine (A) Micro-scale approaches focus on individual node features (e.g., centrality measures). (B) Mesoscale approaches identify functional modules or communities within the network. (C) Macro-scale approaches quantify information flow and characterize the system on a global scale. (D) Multilayer approaches integrate multiple network layers to capture complex interactions across different systems.

**Figure 2.**
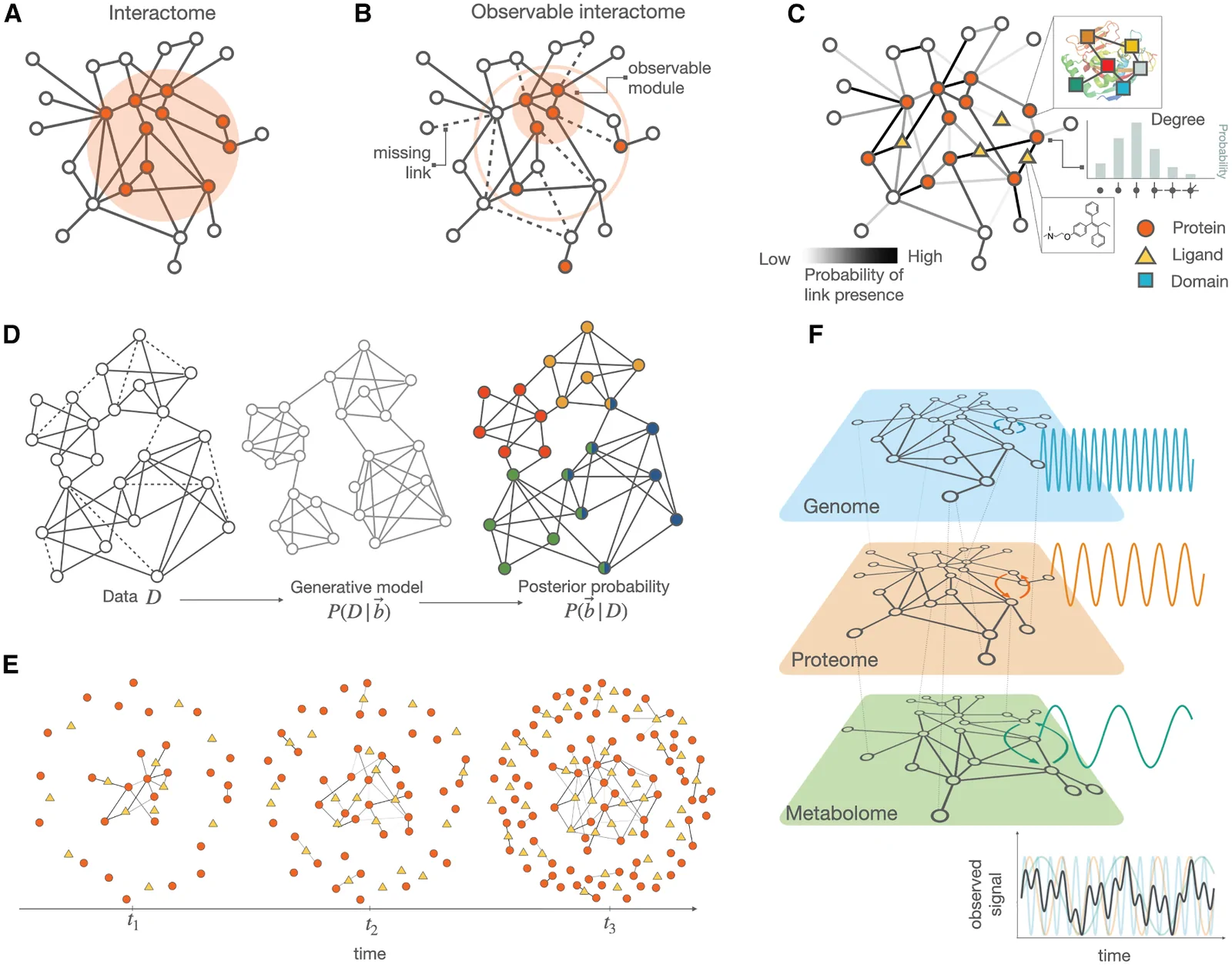
Illustration of different limitations and possible related approaches in network medicine (A) A disease module identified from (within) a full interactome. Nodes associated with a disease are colored (adapted from Menche et al.^37^). (B) The observable disease module is a subset of this module, owing to data incompleteness (adapted from Menche et al.^37^). (C) Physical interaction network in which nodes represent physical proteins and ligands and edges represent their physical associations. In this framework, nodes comprise their constituents (such as protein binding domains, active sites, or interaction interfaces). In a probabilistic network model, each link has a marginal probability of existence, and measures at the level of a single node or the whole network are replaced by probability distributions, encoding how likely it is to measure a specific value.^106^ (D) To characterize different modules in an incomplete or noisy network (D), it is possible to use a nonparametric statistical inference framework that includes the information from a generative model $P(D|\vec{b})$ (in this example, a stochastic block model with B modules, where $b_{i}$ is the module membership of node i). In this construct, the likelihood that the observed network D is generated by a given partition $\vec{b}$ is the posterior distribution $P(\vec{b}|D)$. The inference procedure consists of maximizing the posterior distribution or, as shown in the example, in sampling partitions from the posterior distribution and in considering marginal probabilities of group memberships for each node. (E) In the absence of valid intracellular data on protein or metabolite concentrations or of rate or binding constants, the development of a relevant dynamical representation of biological networks is needed. (F) Integration of multi-omics data needs a novel approach able to capture structure and dynamics within and across sub-systems.

## Box 1. Fundamentals of network science for network medicine

A complex network is a mathematical abstraction used to capture the interactions and relationships (links) among a set of units (nodes). Biological systems of interest for network medicine are gene regulatory networks (GRNs), protein-protein interaction networks (PPINs), metabolic networks (MNs), and cellular communication networks (CCNs).

**Table:**

Concept	Description	Examples
Connectivity	basic property of nodes, denoting the number of connections (degree or degree centrality); connectivity patterns refer to the degree distribution and non-trivial topological correlations	regulatory interactions in GRNs; interactions between enzymes and substrates in MNs^6^; connections between neurons in different brain regions^7^
Centrality	measure to identify crucial biomolecules or genes that play significant roles in the functionality or malfunctioning of biological systems, such as signaling pathways and disease phenotypes; usually adopted for measures more sophisticated than degree	genes with a large number of regulatory connections in GRNs; proteins involved in multiple functions of PPINs
Hubs	nodes with a large degree, often representing key regulatory genes or proteins pivotal in disease pathways essential for cell or organism survival	genes regulating multiple downstream genes in GRNs^8^; proteins that interact with many other proteins in PPINs^9^; high-degree nodes in oncogenic pathways in MNs
Scale-free networks	networks following a scale-free distribution, where a few nodes (genes, proteins, metabolites) have very high connectivity, while the vast majority of nodes have few connections	a few genes regulating several target genes in GRNs, relevant in stress and disease responses; in MNs, a few metabolites are involved in several metabolic reactions, while several metabolites are involved in a few reactions^10^
Motifs and global clustering coefficient	motifs are small and statistically significant sub-systems, involving a few nodes and reflecting fundamental mechanisms; the tendency to form closed triads (motifs of order 3) defines global clustering	proteins in PPINs that form complexes vital in cellular processes and disease mechanisms; genes in GRNs that co-regulate others, forming stable triads significant in maintaining cellular functions^11,12^
Modular and mesoscale organization	tight-knit clusters that might indicate functional modules or common pathways in biological systems crucial for understanding disease mechanisms or finding therapeutic targets	metabolic pathways in MNs^13^; functional brain modules in neural CCNs^14^; rich-club, local, and global integration in NNs^15^
Mixing patterns	tendency of genes or proteins to connect preferentially to nodes with very (dis)similar connectivity	enzymes preferentially interacting with substrates that are similar in MN connectivity; neurons connecting preferentially with others that have similar or dissimilar degrees
Network dynamics	changes of state in biological networks over time or the propagation of effects due to stimuli or disruptions, crucial for understanding disease progression and the impact of therapeutic interventions	gene expression changes over time in GRNs; fluctuations of metabolite concentrations in MN dynamic changes or collective behavior in neural activity^16–18^
Small-world phenomenon	networks with high global clustering and relatively short average path length, related to robustness and efficiency in network communication, crucial for rapid and effective responses to stimuli or disturbances	short pathways in highly clustered GRNs; efficient signaling of neural circuits^19^ in health and disease^20^; metabolites rapidly converted or transported through a few reactions in MNs, enhancing the response to nutritional or environmental changes
Robustness and percolation theory	response of a system to connectivity changes; the theory offers a framework to understand how disruptions (e.g., gene deletions, protein malfunctions) might affect overall biological function; as such, this concept is relevant for understanding disease mechanisms and developing targeted treatments	impact of gene knockout in GRNs; effects of drug interactions on metabolic pathways in MNs; key functional role (e.g., lethality^21^ or essentiality^9^) of proteins in PPINs
Multilayer networks	systems consisting of multiple interconnected or interdependent layers, where each layer represents a different type of interaction or relation	various types of co-occurring protein interactions (e.g., colocalization, physical)^22^; interdependence in host-virus PPINs^23^; multilayer communities^24^ or robustness to perturbations^25^ involving multi-omics and different molecular determinants^26^

## Box 2. Network definitions relevant to network medicine

### Mathematical representation.

The adjacency matrix A is a basic representation of a network, describing the connections between nodes. The elements of A are defined as $A_{ij}=1$ if there is an edge between nodes i and j, and $A_{ij}=0$ otherwise. If the network changes over time, a time-varying adjacency matrix A(t) is used.

### Degree k and degree distribution P(k).

The degree $k_{i}$ of a node i is defined as the number of edges connected to it. Nodal degree is a simple measure used to characterize the connectivity of nodes and can be calculated from the adjacency matrix as $k_{i}=\sum_{j}A_{ij}$, with variants accounting for directionality and the presence of weights. The degree distribution P(k) of the network is then defined as the probability that a randomly selected node has degree k. This is a fundamental characteristic of the topology, and for many biological networks, P(k) is not homogeneous but tends to be fat tailed.

### Generative models of network structure.

Generative models provide a way to understand and predict the structure of networks based on underlying rules.^110^ By using a probabilistic approach, one can express the likelihood of observing a particular network structure A (the model) given some data D (the observation). According to Bayes’ theorem, the posterior distribution of a network model given the observational data is P(A|D)∝P(D|A)P(A). Here, P(A) represents the prior probability of a network structure, and P(D|A) is the likelihood of the data given the network structure. The set of all possible network configurations according to a given model denotes an ensemble. The ensemble can depend on specific constraints (e.g., fixing an average degree) and unknown parameters that can be found by an inferential procedure that maximizes the posterior distribution. Accordingly, the procedure will provide the value of the parameters of the model that better describes the observed data. This procedure is suitable for the analysis of correlation networks, as these comprise the vast majority of datasets built for biological systems. Together with other approaches^106,111^ that map correlation networks into probabilistic ones, this framework can be used to incorporate the experimental uncertainty and mechanistic hypothesis for the inferential process.

### Multilayer network representation.

Multilayer networks extend the classic network structure to accommodate multiple types of interactions or different contexts or *layers*, such as different connectivity patterns. The connectivity within a multilayer network is represented by a multilayer adjacency tensor $M_{j\beta }^{i\alpha }$.^118^ In this tensor, i and j are node indices, while α and β denote layer indices. Each element of this tensor indicates the presence or absence of a connection from node i in layer α to node j in layer β. This formalism allows one to model a broad variety of systems exhibiting multiplexity (i.e., the co-existence of distinct types of interactions among the same set of units) or interdependency (i.e., the interconnection between heterogeneous units, such as microRNA [miRNA]-gene or metabolite-neuronal interactions).^26^

### Dynamics of states in multilayer networks.

Consider a state (e.g., metabolite concentration) $x_{i}(t)$ associated with unit i=1,2,…,N in layer α=1,2,…,L at time t. The time evolution of this state is governed by a set of ordinary differential equations (ODEs) or, in some cases, such as transcription factor binding events, stochastic differential equations (SDEs) that incorporate interactions between nodes as well as node-specific dynamics. A general form of such an ODE might be

(Equation 1)
$$\frac{{dx}_{i\alpha }(t)}{dt}=h_{i}\left[x_{11}(t),x_{12}(t),\ldots ,x_{NL}(t),t\right]\approx f_{i\alpha }\left[x_{i\alpha }(t)\right]+\sigma \sum_{\beta =1}^{L}\sum_{j=1}^{N}M_{j\beta }^{i\alpha }(t)g\left[x_{i\alpha }(t),x_{j\beta }(t),t\right],$$

where $f_{i\alpha }$ represents the intrinsic dynamics of node i in layer α and the second term models the influence of other nodes on the state of node i through two-body interactions within and across layers. The function $g\left[x_{i\alpha }(t),x_{j\beta }(t),t\right]$ specifies the interaction between node i and node j within and across layers, which can vary with time, and $M_{ij}(t)$ is the time-dependent multilayer adjacency tensor, indicating whether a direct interaction between nodes i and j exists at time t.
